# Sulfosalicylic acid to detect proteins in urine of pregnant women

**DOI:** 10.1016/j.mex.2023.102101

**Published:** 2023-03-07

**Authors:** Sandra A. Fernández Macedo, Esperanza Cueva Rossel, Sonia Benita Fernández Tapia, Julio Jimenez Agüero

**Affiliations:** a Nestor Caceres Velasquez Andean University Puno-Peru, Peru; b Manuel Nuñez Butron Hospital Puno-Peru, Peru; c Hospital Santa Rosa Lima-Peru, Peru

**Keywords:** *Sulphosalicylic acid to determine protein in the urine of pregnant women*, Sulfosalicylic acid, Pregnant woman, Proteinuria, Pre-eclampsia

## Abstract

Preeclampsia is one of the main causes of maternal mortality in the Department of Puno, this complication is a hypertensive disease of pregnancy, it is a public health problem worldwide, for this reason it is necessary to make a timely and preventive diagnosis.

An alternative, for the confirmation of this disease, is the rapid detection of proteinuria, using sulfosalicylic acid, which, due to its predictive value, can be used in establishments that do not have personnel or laboratories to carry out clinical examinations.

• It is an easy-to-use reagent that uses the urine of the pregnant woman.
• Support for the diagnosis of preeclampsia in pregnancy
• In addition, it is used to detect proteinuria in systemic diseases.

## Graphical abstract

**Figure:**
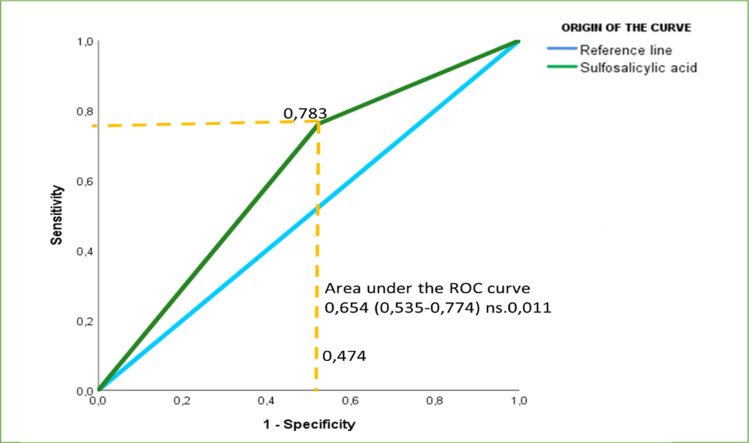

Specifications table

**Table:** 

thematic area:	Pharmacology, Toxicology and Pharmaceutical Science
More specific subject area:	*Microbiology*
Method name:	*Sulphosalicylic acid to determine protein in the urine of pregnant women*
Name and reference of the original method:	*sulfosalicylic acid*
resource availability:	Substance name 5-sulfosalicylic acid dihydrate Molecular formula C₇H₆O₆S 2 H₂O Molar mass 254.2 g /mol REACH Registration No 01–2,120,760,181–63-xxxx CAS No. 5965–83–3 EC—No 641–618–6

## Introduction

3% sulfosalicylic acid applied to the urine of pregnant women seeks to find proteinuria through the degree of turbidity produced by protein denaturation [1]. The absence of protein in the urine is considered negative, it is positive to a cross (+) if the urine is cloudy and not granular with less than 50 mg of protein in urine, turbidity with granules to two crosses (++) with greater proteinuria 200 mg, considerable turbidity and agglutination at three crosses (+++) with proteinuria of 500 mg and a dense cloud with large granules and agglutination that can solidify at four crosses (++++) with proteinuria greater than 500 mg [2].

Highly alkaline urine concentration (density >1030) may show false positives, affecting sensitivity; and very dilute urine (density <1010) alter the specificity, causing false negatives [3].

The objective of the study was to test the efficacy of 3% sulfosalicic acid, its sensitivity and specificity in pregnant women's urine. The test determines the sensitivity, when it detects the disease, it lasts in the probability of correctly classifying a sick individual, that is, that the result is positive for a sick subject [4]. False positives were found when pregnant women did not have the disease and the results were positive; false negatives when they had the disease and the test results were negative [5]. Confirming or rejecting the test determines its usefulness [6]. The best cut-off point for the largest area under the ROC curve corresponds to high sensitivity and specificity [7].

The test also establishes a positive predictive value, (PPV) which is the probability of finding true positives out of the total positive tests and a negative predictive value, (NPV) which is the probability of having negative tests out of the total negative tests [8].

The use of test strips and sulfosalicylic acid is regulated by the Ministry of Health of Peru and is included in the directives of the laboratory network [9]. They have limited applicability in the NETWORK header, it only works to collect samples and process them in the Hospital II clinical laboratory, this delays the diagnosis for a timely referral.

An alternative is the use of 3% sulfosalicylic acid, at the first level of care, due to its easy applicability, low cost, and immediate diagnosis, in its different degrees of turbidity, until reaching proteins ≥300 mg, agglutination in two crosses [10]. under abnormal conditions denatured by this anionic reagent [11], together with arterial hypertension and edema, it gives us the diagnosis of preeclampsia, it can increase during pregnancy and as the weeks of gestation progress [12], If it persists, it produces renal alteration, edema and progresses to chronic kidney disease or eclampsia [13].

Preeclampsia is described, in medical specialty books, as a severe variable multisystem syndrome specific to pregnancy, characterized by a reduction in systemic perfusion generated by vasospasm and activation of the coagulation systems [13], with the presence of systolic blood pressure ≥ 140 mm Hg and/or diastolic ≥ 90 mm Hg in a period of 6 h on at least two occasions, associated with proteinuria ≥ 300 mg from 20 weeks of gestation [14].

## Method details

This research was of an analytical observational type. The participants were 84 pregnant women who had a diagnosis of Preeclampsia, referred from different health centers to the Manuel Núñez Butrón General Hospital in Puno

For sample processing, 24 h urine was collected from pregnant women.

In a test tube, 5 ml of urine was measured plus 3 drops of 3% sulfosalicylic acid, the color change was demonstrated on a black background, and it is interpreted: transparent (without proteinuria), cloudy (there is proteinuria), dense cloud with granules large and agglutination that can solidify greater than 500 mg. determines massive proteinuria of four crosses (++++), the degree of turbidity is directly proportional to the protein concentration according to the number of crosses present [15]. Fig. 2.

The data obtained were tabulated in the free software SPSS. Version 23; determining the sensitivity and specificity, for its validity and the

**Fig. 1:**
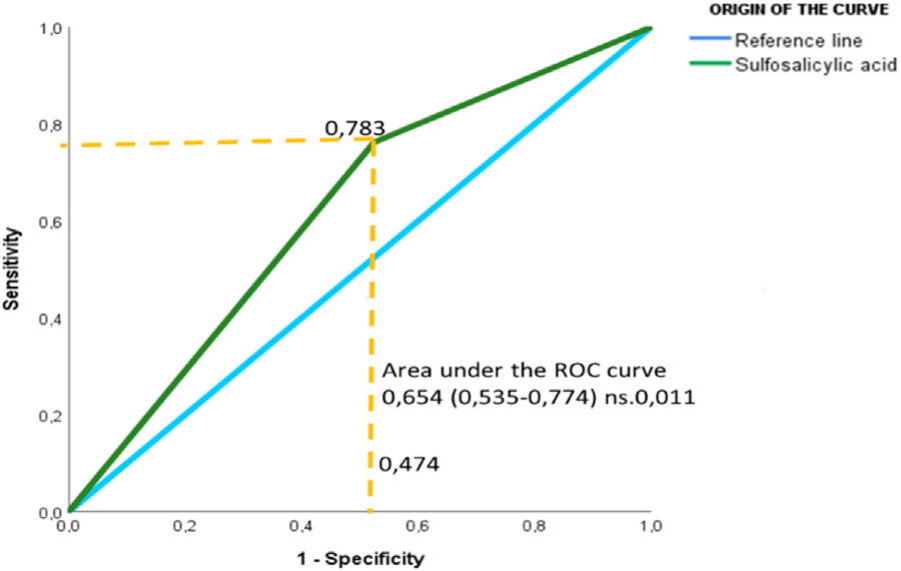
sensitivity and specificity of acetyl sulfasalicylic acid.

Positive Predictive Value and Negative Predictive Value, to see the safety of this diagnostic test, using the Reception Operative Characteristic Curve (ROC) [16]. resulting in a sensitivity of 78.3%, specificity of 47.4%. Fig. 1

**Fig. 2:**
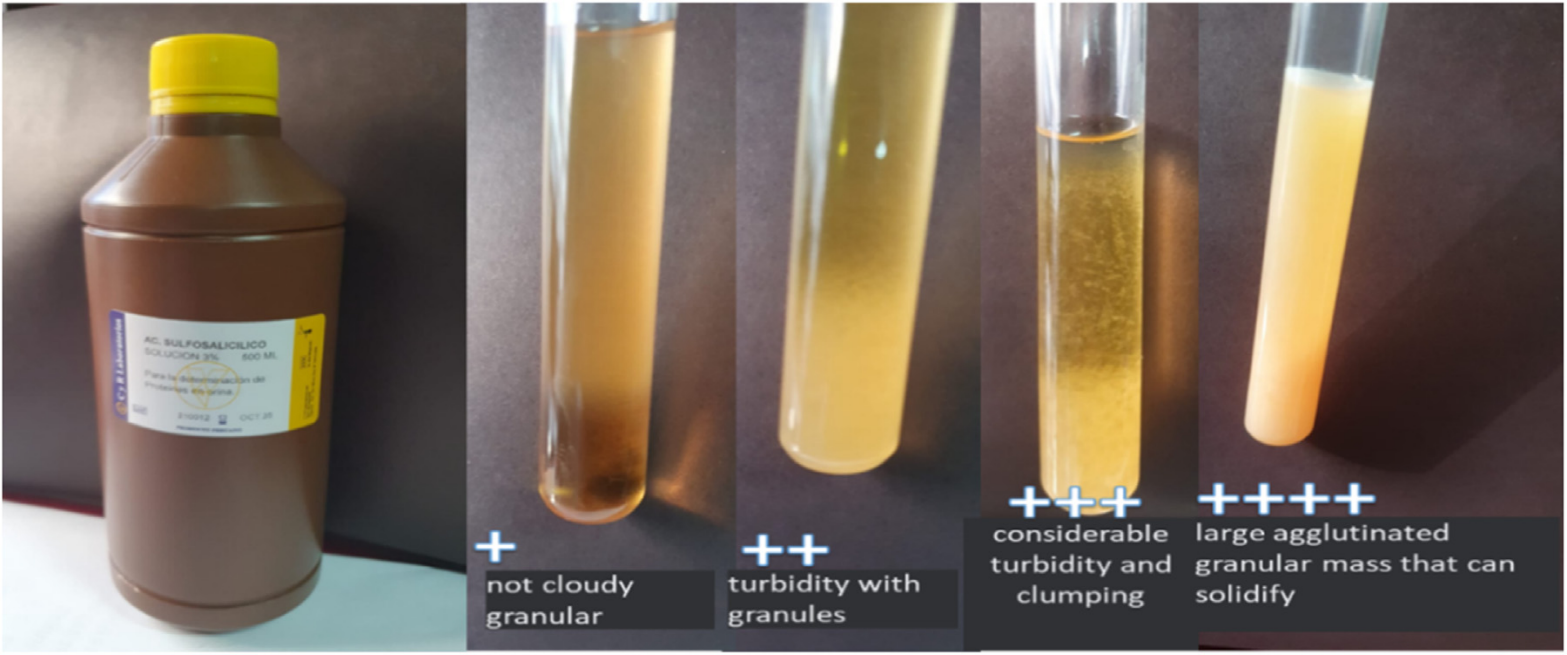
Turbidity classification. The different degrees of turbidity are appreciated by crosses, ranging from one to 4 crosses when urine reacts with sulfosalicylic acid.

## Results

Fig. 1. The light blue color draws a diagonal line that goes from 0 to 1, which is the reference called the 50% discrimination test, between sensitivity and specificity. The green line shows the cut-off point that reaches the highest sensitivity, the yellow line gives us the point of intersection between them, showing us the area under the curve, which is the probability that the test correctly classifies pregnant women diagnosed with preeclampsia.

The area under the curve shows us the ability to correctly discriminate the test, considering a value of 0.654, a confidence interval of 95%, a lower limit of 0.535, an upper limit of 0.774 and a significance level of 0.011. (*p* ≤ 0.05) (Table 1). Which indicates that the test is capable of distinguishing between positives and negatives, that is, the presence of proteinuria using sulfosalicylic acid, this being highly significant.

**Table 1:** Area under the ROC curve.

	**Area under the ROC curve**				
Test outcome variables	Area	Desv. Error	Asymptotic significance	95% asymptotic confidence interval	
				Lower limit	Upper limit
Sulfosalicylic acid	,654	,061	,011	,535	,774

The ROC curve coordinates found a sensitivity of 0.783 and a specificity of 0.474 (Table 2).

**Table 2:** ROC curve coordinates.

**ROC curve coordinates**		
Test outcome variables: Sulfosalicylic acid		
positive if greater than or equal to	Sensitivity	specificity
,00	1000	1000
1,50	,783	,474
3,00	,000	,000

The pregnant women, with a presumptive diagnosis of preeclampsia, were 84 women considered in this trial; (38 Women) 18 were true positives, 20 false negatives;(46 women) 36 were true negatives, and 10 false positives (Table 3).

**Table 3:** False positives and negatives of sulfosalicylic acid and proteinuria.

	Proteinuria > 300 mg urine in 24 h		
	Negative	Positive	Total
Sulfosalicylic acid	Recuento	Recuento	Recuento
Negative	10	20	30
Positive	36	18	54
Total	46	38	84

The sulfosalicylic acid test shows a positive predictive (VPP) value of 47.4% and a negative predictive value (VPN) of 21.7% for 24-hour urine proteinuria (Table 4).

**Table 4:** Positive and negative predictive value of sulfosalicylic acid and proteinuria.

	Protein >300 mg urine 24 hrs					
	(-)		(+)		Total	
Sulfosalicylic acid	n	%	n	%	n	%
Negative	10	21,7%	20	52,6%	30	35,7%
Positive	36	78,3%	18	47,4%	54	64,3%
Total	46	100,0%	38	100,0%	84	100,0%

Sensitivity is based on the probability that a sick person is actually sick [17]. That is, when 5 mL of the pregnant woman's urine was taken, it tested positive for preeclampsia, thus the ability of the acid test to detect the disease. The pregnant women who underwent the urine sulfosalicylic acid test were diagnosed regardless of the number of pregnancies, since these are risk factors for the appearance of preeclampsia [18], however, the faster its diagnosis and transfer is made, the better its prognosis will be since Puno is the fifth Department of Peru with the highest maternal mortality due to direct and indirect deaths [19].

The positive results in the 24-hour urine of the pregnant woman show that this reagent can accurately detect preeclampsia due to its low cost and applicability, it should be used in all health establishments; Compared to expensive tests in clinics and private laboratories, such as albumin/creatinine tests or creatinine index [20]. for this pregnancy complication and that not all pregnant women can access it due to their poor economy.

## Conclusion

Sulfosalicylic acid confirmed the presence of proteinuria in 24 h urine, with a significance level of 0.000, forming a precipitate of 3 crosses (considerable turbidity and agglutination) and 4 crosses (dense cloud with a large agglutinated granular mass) in pregnant women diagnosed with of preeclampsia, with a sensitivity of 78.3%, a specificity of 47.4%, a Positive Predictive Value (PPV) of 47.4% and a Negative Predictive Value (NPV) of 21.7%.

## Declaration of Competing Interest

The authors declare that they have no known competing financial interests or personal relationships that could have appeared to influence the work reported in this article.

## Data Availability

Data will be made available on request.
